# The Memphis School

**Published:** 1850-10

**Authors:** 


THE MEMPHIS SCHOOL.

The Western Lancet denounces Dr. James C. Cross for his connection with the Memphis School; we cannot join in this denunciation, because we think Dr. Cross may be safely left to the enjoyment of his position. B.

